# Book Reviews

**Published:** 1900-12

**Authors:** 


INTERNATIONAL CLINICS. Vol. 3. 10th Series. Price $2.00 per vol. J. B. Lippincott Company, Philadelphia.

This series is now edited by Henry W. Cattell, with the collaboration of John B. Murphy, H. C. Wood, T. G. Morton, T. M. Rotch, Charles H. Reed, A. D. Blackader and A. Landolt, a new corps of editors. The present volume contains interesting articles. L. Duncan Bulkley on Extra-genital Chancre, Gottheil on some of the Tertiary Accidents of Syphilis, and others on genito-urinary subjects. There is a fine illustrated article on injuries of the eyelids and eyeballs, by L. Webster Fox, and two extended contributions by Wm. Kraus on the use and care of the microscope, and Thomas S. Westcott on the scientific modification of milk. The book shows the usual typographical excellence of Lippincott's publications.

---

ANATOMY AND HISTOLOGY OF THE MOUTH AND TEETH. J. Norman Broowell, D.D.S. P. Blakiston's Son & Co. Price $4.50.

This work consists of a systematic arrangement and classification of the structures concerned in dental work. Besides this there are special chapters on special details of dental and buccal morphology. The book is elaborately illustrated, most of the illustrations being reproductions of photographs taken by the author from nature. The work for its completeness and painstaking thoroughness is sure to be appreciated by the dental profession.

THE ANNUAL OF ECLECTIC MEDICINE AND SURGERY. Edited by John V. Stevens, M.D. Vol. 8, embracing the papers and proceedings of the various State Eclectic Medical Societies for the years 1897 and 1898. 8 vol., 538 pp. Cloth, price $2.00. The Scudder Brothers Company, Publishers, Cincinnati, Ohio, 1900.

In this volume a large number of papers on a great variety of subjects are collected. For the most part the articles are well written and many deal with subjects of general interest to the medical profession without school restriction. The Eclectics are doing good work and there is much that is commendable in their teachings.

---

THE MICROTOMIST'S VADE MECUM. A Handbook of the Methods of Microscopic Anatomy. By Arthur Bolles Lee. Price $4.00. P. Blakiston's Son & Co., 1012 Walnut street, Philadelphia. 1900.

This handbook contains general and specific information on all subjects in the field of medical microscopy. The subject-matter is condensed, but not to the sacrifice of clearness. It would be found valuable not only as a working gnide but also for a reference book to keep in the library for consulting when some formula or method in microscopic technic is desired.

---

A REVIEW OF RECENT LEGAL DECISIONS AFFECTING PHYSICIANS, DENTISTS, DRUGGISTS AND THE PUBLIC HEALTH. By W. A. Purrington, of the New York Bar. E. B. Treat & Co., New York. 1899.

W. A. Purrington is counsel for the New York County Medical Society. He has given an abstract of court decision bearing on the various relations of doctor and dentist to the public. The volume includes an essay on manslaughter.

---

HYGIENE AND SANITATION. Lea Bros. & Co., New York and Philadelphia.

---

A TEXT–BOOK OF HISTOLOGY. Böhm, Davidoff, Huber. Price $3.50. W. B. Saunders & Co., Philadelphia and London.

---

A MANUAL OF MATERIA MEDICA AND PHARMACOLOGY. Culbreath. Lea Bros. & Co. New York and Philadelphia.

